# A duplex real-time reverse transcriptase polymerase chain reaction assay for detecting western equine and eastern equine encephalitis viruses

**DOI:** 10.1186/1743-422X-7-284

**Published:** 2010-10-26

**Authors:** Xiaoping Kang, Yuchang Li, Hong Liu, Fang Lin, Xuyu Cai, Tingting Sun, Guohui Chang, Qingyu Zhu, Yinhui Yang

**Affiliations:** 1 State Key Laboratory of Pathogen and Biosecurity, Beijing Institute of Microbiology and Epidemiology, Beijing 100071, China

## Abstract

In order to establish an accurate, ready-to-use assay for simultaneous detection of Eastern equine encephalitis virus (EEEV) and Western equine encephalitis virus (WEEV), we developed one duplex TaqMan real-time reverse transcriptase polymerase chain reaction (RT-PCR) assay, which can be used in human and vector surveillance. First, we selected the primers and FAM-labeled TaqMan-probe specific for WEEV from the consensus sequence of NSP3 and the primers and HEX-labeled TaqMan-probe specific for EEEV from the consensus sequence of E3, respectively. Then we constructed and optimized the duplex real-time RT-PCR assay by adjusting the concentrations of primers and probes. Using a series of dilutions of transcripts containing target genes as template, we showed that the sensitivity of the assay reached 1 copy/reaction for EEEV and WEEV, and the performance was linear within the range of at least 10^6 ^transcript copies. Moreover, we evaluated the specificity of the duplex system using other encephalitis virus RNA as template, and found no cross-reactivity. Compared with virus isolation, the gold standard, the duplex real time RT-PCR assay we developed was 10-fold more sensitive for both WEEV and EEEV detection.

## Introduction

Eastern equine encephalitis virus (EEEV) and western equine encephalitis virus (WEEV) are both arthropodborne viruses, belonging to the genus *Alphavirus*, family Togaviridae. Both viruses are mainly spread in America and transmitted by mosquitoes to equines, birds and humans, causing a febrile disease (including encephalitis) with a significant frequency of fatal outcomes. The fatality rate of EEEV is approximately 30-70% [1,2], the most severe one among the arboviral encephalitis; while the fatality rate of WEEV is approximately 3%. Although there is an obvious difference in case fatality rate between the two species, they share a high similarity in genome sequence and antigenicity. In order for better rescue and precaution procedures for WEEV and EEEV infection, it is crucial to develop rapid and accurate methods to detect and discriminate these two species.

Compared with traditional methods such as RT-PCR, IFA, ELISA and virus isolation, real-time RT-PCR has the advantage of fast speed and improved sensitivity. Therefore, it has been developed quickly and become the main method for pathogen detection[3]. Real-time RT-PCR has the ability to measure several fluorophores in one well and permits multiplex assays, so it can be used to detect different target sequences simultaneously in one reaction [4].

In this study, we designed the primers and probes of real-time RT-PCR for EEEV and WEEV, and we have established a duplex real time RT-PCR method for simultaneously detection and discrimination of these two viruses.

## Materials and methods

### Primer and probe design

The primers and probes for EEEV and WEEV detection were designed by software SDS2.0, which were list in table 1. The target sequences were selected from the conserved regions of E3 gene for WEEV and NSP3 gene for EEEV, respectively. The specificity of the primers and probes was confirmed by BLAST in Genbank, the matched sequences in other species were less than 50%. The probe for EEEV was labeled with the reporter HEX at the 5' end and TAMRA at the 3' end, and the probe for WEEV was labeled with the reporter FAM at the 5' end and TAMRA at the 3' end.

**Table 1:** Primer and probe sequences for the duplex real-time RT-PCR for EEEV and WEEV

Name of primer or probe	Sequence (5'→3')	Nucleotide start
EEE-F	TGTGCGTACCTCCTCATCGTT	335
EEE-R	GACTGGCGTGAATCTCTGCTT	414
EEE-Probe	HEX-AGCAGCCTACCTTTCCGACAATGGTTGTC-TAMRA	364
WEE-F	AGGGATACCCCCGAAGGTT	8220
WEE-R	GTGAATAGCACACGGGTGGTT	8322
WEE- Probe	CTTTCGAATGTCACGTTCCCATGCG	8274

### Virus culture and isolation

WEEV (McMillan stain) and EEEV (SSP. North American variant) were cultured in BHK-21 cells, and the supernatants were collected after cytopathic effect (CPE) appeared. Virus isolation was conducted by cell culture. BHK cells were inoculated with different dilutions (a series of dilution of 1-10^10^) of WEEV and EEEV and incubated at 37°C, and CPE was observed after 4 days. All virus isolations were performed in quadruplicate. The titres of WEEV and EEEV were determined by tissue culture infective dose per ml (TCID_50_/ml). The titre was 2 × 10^8 ^TCID_50_/ml for EEEV and 5 × 10^8 ^TCID_50_/ml for WEEV, respectively.

Reference strains in this study included Chikgunya virus (CHIK), Japanese B encephalitis virus (JEV), Tick born encephalitis virus (TBEV), St. Louis encephalitis virus (SLEV), Dengue virus (DEN) and Yellow fever virus (YFV).

### Nucleic acid extraction

RNA was extracted from the supernatants of cultured viruses by RNeasy mini kit (Qiagen Inc., Valencia, CA, USA) according to the manufacture's instructions. All the RNA extraction procedure was conducted at BSL-3 laboratory.

### In vitro transcription of plasmid DNA

Using linearised plasmid DNA containing the target sequence, RNA was transcribed in vitro with the Riboprobe(r) System-SP6/T7 (Promega, USA) according to the manufacturer's instructions. The T7-transcribed positive control was digested with DNase and purified with the RNeasy Kit (Qiagen, USA). The correct size of the transcribed RNA was confirmed by formaldehyde agarose gel electrophoresis, and the concentration was determined by spectrophotometry. The stock solutions of the in vitro-transcribed RNA were stored at -70°C, and the diluted working solutions were stored at -20°C[5].

### Sensitivity and specificity of the duplex real-time RT-PCR assay

The sensitivity of the duplex real-time RT-PCR was conducted as follows: the reaction consisted of 10 μL 2 × reaction buffer, 0.2 μL reverse transcription enzyme, 250 nmol/L primers for WEEV and 500 nmol/L for EEEV, 150 nmol/L probes for WEEV and 250 nmol/L for EEEV. In the analysis, 2 uL serial diluted transcript RNA of EEEV or WEEV (0.01-10^6 ^copies, 10-fold diluted) was added into the tubes as template, deionized water were added to the final volume of 20 uL. PCR was performed with a LightCycler 2.0 (Roche, Switzerland). The reactions were incubated at 50°C for 30 min, followed by 95°C for 10 min (inactivation reverse transcriptase/activation Taq polymerase), 45 cycles of 95°C for 15 s (denaturation), and 55°C for 1 min (annealing). The fluorescence emitted from the assay was captured during the annealing phase of each cycle at 530 nm for WEEV and 560 nm for EEEV, respectively. The results were analyzed with LightCycler software (version 4.05). The RNAs of reference strains were also tested with this assay in order to examine the assay specificity [6-8].

### Detection of the mimic samples by the duplex real-time RT-PCR

The mimic samples were prepared by mixing the virus with the mouse brain tissue, then the brain tissues were grinded, and the RNA was extracted and applied with the duplex real-time RT-PCR assay.

## Results

### Sensitivity and specificity analysis of the duplex system

**Figure 1:**
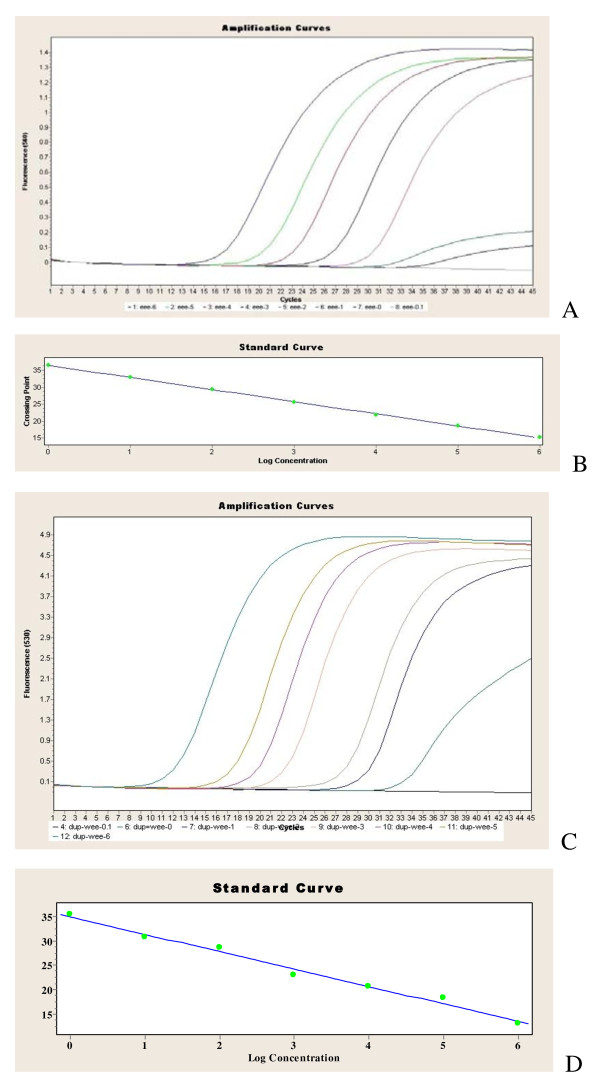
**Analytical sensitivity of the duplex real time RT-PCR assay based on the 10-fold dilution series of the in vitro-transcribed RNA**. The amplification blot for EEEV(A) and the associated standard curve graph (B)for EEEV, The amplification blot for WEEV(A) and the associated standard curve graph (D)for WEEV, are all depicted.

Figure 1 and Table 2 show the sensitivity of the duplex system: the detection limit reached 1 copy/reaction for both EEEV and WEEV. Moreover, the performance was linear for at least 10^6 ^transcript copies. The positive signals only appeared in one default channel, 530 nm for WEEV and 560 nm for EEEV, indicating no cross reaction between the two species in the duplex system.

**Table 2:** Sensitivity of the real-time RT-PCR assay and virus isolation

Virus	Dilution	Virus isolation	Real time RT-PCR	CT
WEEV	10^-1^	4/4	+	15.25
	10^-2^	4/4	+	18.29
	10^-3^	4/4	+	22.02
	10^-4^	4/4	+	25.49
	10^-5^	4/4	+	29.28
	10^-6^	2/4	+	32.79
	10^-7^	0/4	+	36.03
	10^-8^	0/4	-	

TCID_50_: 10^6^/0.1 ml				

EEEV	10^-1^	4/4	+	11.37
	10^-2^	4/4	+	16.25
	10^-3^	4/4	+	18.54
	10^-4^	4/4	+	22.03
	10^-5^	4/4	+	27.35
	10^-6^	2/4	+	30.18
	10^-7^	1/4	+	34.79
	10^-8^	0/4	+	37.59

TCID_50_: 10^6.25^/0.1 ml				

To evaluate the cross-reaction with other encephalitis viruses, a panel of six viruses including JEV, TBEV, CHIK, DEN, YFV and SLEV were tested. All the samples appeared to be negative and only background fluorescence levels were observed (data not shown), demonstrating that our real-time RT-PCR detection system was specific for EEEV and WEEV.

### Sensitivity of the duplex system compared to virus isolation

For a comparative study of the duplex real-time RT-PCR system and the virus isolation, a serial of 10-fold diluted culture supernatants of WEEV and EEEV were prepared. One aliquot of each dilution was used for virus isolation, and a second aliquot was used for RNA extraction and duplex real-time RT-PCR analysis. Table 2 demonstrates that the duplex real-time RT-PCR assay was 10-fold more sensitive than virus isolation for both WEEV and EEEV detection.

### Detection of the mimic samples

The mimic samples were prepared by mixing the virus with the mouse brain tissue, and then used for real-time RT-PCR detection. The positive results for EEEV or WEEV was obtained. The assay was also tested with the virus mixture containing both EEEV and WEEV, and the positive signals were appeared both at 530 nm and 560 nm, indicating that this duplex system is suitable for detecting both EEEV and WEEV infection.

## Discussion

Antibodies against EEEV has been found in the serum of some encephalitis patients in China by using immunofluorescence assay [9,10], but no EEEV and WEEV strains have been isolated so far. Due to the cross-reaction among *Alphavirus *by immunoassay, it remains unclear whether EEEV infection exits in China. With the globalization of international trade, the rapid and free movement of large amounts of people, animals, food and feed products has created the risk of a novel and epidemiologically vulnerable situation. Infectious agents have a chance to spread all over the world within several hours or days. In order to better control import infection and monitor the prevalence of encephalitis diseases in China, it is urgent to develop rapid and accurate methods for WEEV and EEEV detection.

There are several widely used methods for arboviral pathogen detection including indirect hemagglutination inhibition (IHI), ELISA, virus isolation and RT-PCR. However, each method has its own disadvantages: serologic tests have the limit of cross-reaction[11], and virus isolation is time-consuming and requires biosafety level-3 containment. Reverse-transcriptase PCR (RT-PCR) is a rapid and sensitive method that is being increasingly used as an adjunct to serology for arbovirus diagnosis[11,3]. Real-time RT-PCR is more sensitive and specific, so it is a preferred method for pathogen detection.

Some assays for WEEV and EEEV diagnostics have been developed. Hull et al. developed a duplex real-time RT-PCR assay for EEEV and St. Louis virus[12]; Carrera et al. developed a multiple RT-PCR for 13 arboviruses[5]. Eshoo developed an RT-PCR assay for alphavirus detection[13]. Pässler et al. developed a detecting assay for alphaviruses based on antibodies[14]. Lambert et al. developed an RT-PCR assay and Taqman RT-PCR assay for WEEV and EEEV[11]. But none of the above methods was available for detecting and distinguishing WEEV and EEEV in a single reaction.

In this study, we developed and validated a duplex real-time RT-PCR system for both WEEV and EEEV detection at the same time. The primers and probes were selected from E3 gene for WEEV and NSP3 gene for EEEV, and the sequence analysis confirmed the specificity for WEEV and EEEV, respectively. Using other encephalitis viruses RNA (e.g., DEN, CHIK, TBE and JEV) as template, no cross-reaction signals were detected, demonstrating the specificity of the duplex system.

Using the same concentrations of the primers and probes for EEEV and WEEV, the sensitivity of the duplex real-time RT-PCR system was similar for WEEV and was 20-fold lower for EEEV compared with the single system. In order to improve the sensitivity of the duplex system, we further optimized the concentrations of the primers and probes for EEEV. First, the assay was conducted with different primer concentrations (125, 250, 375, 500, 625 and 750 nmol/L), and the template was a serial of diluted EEEV RNA transcripts. Second, the optimal probe concentrations were determined with different probe concentrations (100, 150, 200, 250 and 300 nmol/L). The most proper primer/probe concentration was achieved by reaching the lowest Ct at the fixed amount of template.

Our results showed that the optimal EEEV primer concentration was 500 nmol/L each and the optimal EEEV probe concentration was 250 nmol/L; the optimal WEEV primer concentration was 250 nmol/L each and the optimal WEEV probe concentration was 150 nmol/L. With these optimized parameters, the assay finally reached 1copy/reation for both WEEV and EEEV.

Furthermore, we compared the duplex RT-PCR system with virus isolation, the 'gold standard' in virus diagnostics. The results showed that our duplex real-time RT-PCR was more sensitive than cell culture isolation. Taken together, the duplex real-time RT-PCR system we developed is a robust and valuable tool for highly sensitive and specific detection of WEEV and EEEV infection.

## Competing interests

The authors declare that they have no competing interests.

## Authors' contributions

XK: designed the study, did laboratory testing, analysed the test results, co-wrote and edited the manuscript. YL, H L, FL, XC, TS and GC took samples and did laboratory testing. QZ and YY organized the overall project and helped edit the manuscript. All the authors read and approve the final manuscript.

## References

[B1] Brault AC Powers AM Chavez CLV Lopez RN Cachon MF Gutierrez LFL Kang W Tesh RB Shope RE Weaver SC Genetic and antigenic diversity among eastern equine encephalitis viruses from North, Central and South America Am J Trop Med Hyg 1999 61 579 586 10548292 10.4269/ajtmh.1999.61.579

[B2] Deresiewicz RL Thaler SJ Hsu L Zamani AA Clinical and neuroradiographic manifestations of eastern equine encephalitis N Engl J Med 1997 336 1867 1874 10.1056/NEJM199706263362604 9197215

[B3] Linssen B Kinney RM Aguilar P Russell KL Watts DM Kaaden OR Pfeffer M Development of reverse transcription-PCR assays specific for detection of equine encephalitis viruses J Clin Microbiol 2000 38 1527 1535 10747138 10.1128/jcm.38.4.1527-1535.2000 PMC86482

[B4] Xiao-Ping KANG Yong-Qiang LI Sun Qing-Ge Hong LIU Yin-Hui YANG Qing-Yu ZHU Development of a consensus microarray method for identification of some severe infective viruses Journal of Medical Virology 2009 81 1945 1950 10.1002/jmv.21602 19774692 PMC7166427

[B5] Carrera M Sagripanti JL Non-infectious plasmid engineered to simulate multiple viral threat agents J Virol Methods 2009 159 29 33 10.1016/j.jviromet.2009.02.021 19442841

[B6] Lambert AJ Martin DA Lanciotti RS Hoffmann K, Depner H, Schirrmeier, Beer M A universal heterologous internal control system for duplex real-time RT-PCR assays used in a detection system for pestiviruses B Journal of Virological Methods 2009 136 200 209 10.1016/j.jviromet.2006.05.020 16806503

[B7] Hoffmann B Beer M Schelp C Schirrmeier H Depner KM Validation of a real-time RT-PCR assay for sensitive and specific detection of classical swine fever Journal of Virological Methods 2005 130 36 44 10.1016/j.jviromet.2005.05.030 16055202

[B8] Xiaoping Kang Tao Jiang Yongqiang Li Fang Lin Hong Liu Guohui Chang Qingyu Zhu Chengfeng Qin Yinhui Yang A duplex real-time RT-PCR assay for detecting avian influenza virus H5N1 and novel influenza virus H1N1 Virology Journal 2010 7 114 10.1186/1743-422X-7-114 20515509 PMC2892456

[B9] Li Chen Guodong Liang Boquan Chen Qiping Li Ying He Liting Song Zijiang Zhao Yijun Huang Detection of antibodies against Sindbis virus and eastern equine encephalitis virus from human serum in China Chinese Journal of Experimental and Clinical Virology 1994 8 4 371 371 (In Chinese)

[B10] Qiping Li Guodong Liang Jichu Xie Isolation of primarily identification of eastern equine encephalitis virus Chinese Journal of Experimental and Clinical Virology 1992 6 1 58 59 (In Chinese)

[B11] Lambert AJ Martin DA Lanciotti RS Detection of North American Eastern and Western equine encephalitis viruses by nucleic acid amplification assays J Clin Microbiol 2003 41 379 385 10.1128/JCM.41.1.379-385.2003 12517876 PMC149608

[B12] Hull R Nattanmai S Kramer LD Bernard KA Tavakoli NP A duplex real-time reverse transcriptase polymerase chain reaction assay for the detection of St. Louis encephalitis and eastern equine encephalitis viruses Diagn Microbiol Infect Dis 2008 62 272 9 10.1016/j.diagmicrobio.2008.07.004 18715737 PMC2615585

[B13] Eshoo MW Whitehouse CA Zoll ST Massire C Pennella TT Blyn LB Sampath R Direct broad-range detection of alphaviruses in mosquito extracts Virology 2007 368 286 95 10.1016/j.virol.2007.06.016 17655905

[B14] Pässler S Pfeffer M Detection of North American eastern and western equine encephalitis viruses by nucleic acid amplification assays J Clin Microbiol 2003 41 379 85 10.1128/JCM.41.1.379-385.2003 12517876 PMC149608

